# Prolonged *Leptospira* Urinary Shedding in a 10-Year-Old Girl

**DOI:** 10.1155/2012/169013

**Published:** 2012-03-25

**Authors:** Emilie Chow, Jaime Deville, Jarlath Nally, Michael Lovett, Karin Nielsen-Saines

**Affiliations:** ^1^The Division of Pediatric Infectious Diseases, Department of Pediatrics, University of California, P.O. Box 951752, Los Angeles, CA 90095-1752, USA; ^2^The Division of Infectious Diseases, Department of Internal Medicine, University of California, P.O. Box 951752, Los Angeles, CA 90095-1752, USA

## Abstract

We present a case of leptospirosis in a previously healthy girl following a trip to Costa Rica. While she was clinically asymptomatic, she had spirochetes cultured from her urine six weeks following her trip. Prolonged urinary shedding following infection with *Leptospira* is possible in humans and often has subtle manifestations in children.

## 1. Case Presentation

A 10-year-old previously healthy girl with no prior remarkable medical history presented to our institution with fever following a one-week trip to Costa Rica. During this time, she stayed in a hotel in the rain forest where she took hikes with family members. She swam in the ocean as well as in chlorinated pools, saw many animals (including rodents), and was splashed with fresh water. Five days following her return from Costa Rica, she developed fever of one-week duration (up to 39.5°C), abdominal pain, nausea, and sore throat. None of the accompanying family members (parents and sibling) developed any signs of illness, nor was there any remarkable family history. The family resided in a metropolitan area in Los Angeles County, and there had been no recent floods or rodent exposures in the neighborhood preceding or following the trip. The patient was seen in her primary care physician's office one week into her illness. At that time, she had a negative throat beta streptococcus direct antigen screen. She developed bilateral eye pain and redness and was seen again by her primary care pediatrician two days later who performed further laboratory evaluations. Results included an alanine aminotransferase of 102 U/L, aspartate aminotransferase of 73 U/L, albumin 3.1 g/dL, total bilirubin 0.5 mg/dL, prothrombin time 12.9 seconds, partial thromboplastin time 38.1 seconds, and alkaline phosphatase 314 U/L.

She was subsequently referred to our infectious disease clinic for further investigation. The first visit to our clinic was approximately 2 1/2 weeks following the return from Costa Rica. At that time, she was afebrile and showed complete symptomatic improvement. Further workup demonstrated a positive *Leptospira* indirect hemagglutination test (Focus technologies, Cypress, CA) with a titer of 1 : 200. After the results of the positive *Leptospira* test became available, she was reevaluated at our clinic (approximately 6 weeks following her return from Costa Rica). At that time, she was back to her normal state of health. A urine culture for *Leptospira* was obtained and incubated onto Ellinghausen McCullough Johnson Harris medium with 200 micrograms per mL of 5-Fluorouracil. Urine cultures 6 weeks following her trip were positive for spirochetes. A renal ultrasound was normal, while serum creatinine and blood urea nitrogen values remained within normal range during this period. A convalescent serology was also obtained. Both acute and convalescent sera were sent to the CDC for confirmation and microscopic agglutination testing (MAT).

Acute and convalescent sera were positive with IgM Dip-S-Ticks (PanBio INDX, Inc., Baltimore, Maryland). The MAT test was negative for the acute serum. The convalescent serum had a positive MAT titer of 1 : 400 to serogroup Mini and serovar Georgia and strain LT117. The WHO/FAO Collaborating Centre for Reference and Research on Leptospirosis, Royal Tropical Institute (KIT, Amsterdam, The Netherlands) has identified the *Leptospira *spirochete as belonging to serovar Shermani using rabbit polyclonal sera. The patient was seen again 2 months following her trip to Costa Rica. She was doing well, and urine cultures for* Leptospira *were performed at the time with negative results.

## 2. Discussion

We present a case of pediatric leptospirosis with recovery of the organism in urine after clinical resolution of disease. Leptospirosis is a worldwide zoonotic disease [1]. Humans become infected when exposed to the urine of infected animals and to urine-contaminated water. Human disease can range from nonspecific manifestations of fever and myalgias to fatal pulmonary hemorrhage, jaundice, and renal failure. Outbreaks of leptospirosis in Costa Rica have been reported in white-water rafters [2]. Our patient was exposed during her trip to Costa Rica and developed anicteric leptospirosis and was able to control symptoms of her infection without antimicrobials. There were no other reported environmental risk factors in this patient's history, and the child had no history of prior infections which would suggest any underlying predisposition or immune deficiency.

Leptospirosis as a nonspecific clinical illness similarly renders nondiagnostic routine laboratory test results. Our patient demonstrated a moderate increase in liver function tests during the acute phase of illness; however, laboratory findings were nonspecific. Following a high level of suspicion, most cases of leptospirosis are diagnosed serologically, with the macroscopic agglutination test (MAT) being the gold standard, although indirect hemagglutination and ELISA assays are also available. In our patient, we had a positive screening serologic test by hemagglutination confirmed by the MAT assay performed at the CDC. Although the patient's acute sera by MAT was negative, the presence of a positive convalescent titer by MAT and the growth of organisms in urine confirmed the diagnosis. Antibody titers found in MAT are not useful to predict the serovar infecting individual patients. Nevertheless, using rabbit polyclonal sera, serovar Shermani was identified as the etiologic agent in this specific case by a WHO referral laboratory in the Netherlands.

In children, the diagnosis of leptospirosis is underappreciated due to its nonspecific presenting symptoms and the lack of rapid and sensitive diagnostic testing for the initial phases of infection. In Hai Yat, Thailand, 27.2% of children had leptospirosis as an etiology of fever following floods [3]. In Mumbai, India, 34% of children presenting with fever after flooding had serologic evidence of leptospirosis [4]. A serologic survey of school children in Trinidad and Barbados revealed 12.5% of Barbadian children, and 9.5% of Trinidadian children sera were positive with MAT testing [5]. Children infected with *Leptospira* can present with fever, myalgia, headache, abdominal pain, hepatomegaly, icterus, acute renal failure, and meningitis [6–8]. Children have a lower in-hospital case-fatality rate of leptospirosis as compared to adults [9]. Pediatric deaths due to leptospirosis have been attributed to ARDS and pulmonary hemorrhage with respiratory failure [7, 8]. Treatment of choice is generally intravenous penicillin or ceftriaxone [10].

 Animals are the main reservoir of infection. Rats [11, 12], dogs [13], mice [14], cows, and other domesticated animals [15] can have chronic renal infection with* Leptospira *and shed the spirochete in their urine and infect humans. Most human cases are diagnosed with serological methods rather than culturing blood and urine for *Leptospira* spirochetes onto selective media. As a result, prolonged urinary shedding in humans has not been well documented. Spirochetes can be detected in the blood during early infection [16, 17]. Reports that *Leptospira* can be cultured from the urine weeks to months after disease are very rare [16]. Using PCR, *Leptospira* has been demonstrated in human urine samples a year following the acute phase of infection [18].

 Our patient illustrates that children can develop prolonged urinary shedding of spirochetes despite resolution of clinical symptoms. Therefore, theoretically humans could act as carriers of *Leptospira* and be the source of infection.

Leptospirosis is an emerging zoonosis that should be considered in the differential diagnosis of febrile pediatric patients who have recently traveled to an endemic area or are exposed to seasonal floods.

## References

[1] Bharti AR, Nally JE, Ricaldi JN (2003). Leptospirosis: a zoonotic disease of global importance. *Lancet Infectious Diseases*.

[2] (1997). Outbreak of Leptospirosis among White-Water Rafters—Costa Rica, 1996. *Morbidity and Mortality Weekly Report Surveillance Summaries*.

[3] Pradutkanchana J, Pradutkanchana S, Kemapanmanus M, Wuthipum N, Silpapojakul K (2003). The etiology of acute pyrexia of unknown origin in children after a flood. *Southeast Asian Journal of Tropical Medicine and Public Health*.

[4] Karande S, Bhatt M, Kelkar A, Kulkarni M, De A, Varaiya A (2003). An observational study to detect leptospirosis in Mumbai, India, 2000. *Archives of Disease in Childhood*.

[5] Everard COR, Hayes RJ, Edwards CN (1989). Leptospiral infection in school-children from Trinidad and Barbados. *Epidemiology and Infection*.

[6] Karande S, Kulkarni H, Kulkarni M, De A, Varaiya A (2002). Leptospirosis in children in Mumbai slums. *Indian Journal of Pediatrics*.

[7] Rajajee S, Shankar J, Dhattatri L (2002). Pediatric presentations of Leptospirosis. *Indian Journal of Pediatrics*.

[8] Marotto PCF, Marotto MS, Santos DL, Souza TNL, Seguro AC (1997). Outcome of leptospirosis in children. *American Journal of Tropical Medicine and Hygiene*.

[9] Lopes AA, Costa E, Costa YA (2004). Comparative study of the in-hospital case-fatality rate of leptospirosis between pediatric and adult patients of different age groups. *Revista do Instituto de Medicina Tropical de Sao Paulo*.

[10] Panaphut T, Domrongkitchaiporn S, Vibhagool A, Thinkamrop B, Susaengrat W (2003). Ceftriaxone compared with sodium penicillin G for treatment of severe leptospirosis. *Clinical Infectious Diseases*.

[11] Vinetz JM, Glass GE, Flexner CE, Mueller P, Kaslow DC (1996). Sporadic urban Leptospirosis. *Annals of Internal Medicine*.

[12] Sarkar U, Nascimento SF, Barbosa R (2002). Population-based case-control investigation of risk factors for leptospirosis during an urban epidemic. *American Journal of Tropical Medicine and Hygiene*.

[13] Barkin RM, Glosser JW (1973). Leptospirosis: an epidemic in children. *American Journal of Epidemiology*.

[14] Matthias MA, Levett PN (2002). Leptospiral carriage by mice and mongooses on the Island of Barbados. *West Indian Medical Journal*.

[15] Faine S, Adler B, Bolin C, Perolat P (1999). *Leptospira and Leptospirosis*.

[16] Berman SJ, Tsai CC, Holmes K (1973). Sporadic anicteric leptospirosis in South Vietnam: a study in 150 patients. *Annals of Internal Medicine*.

[17] Kurtoğlu MG, Tuncer O, Bozkurt H (2003). Report of three children with leptospirosis in rural area of the East of Turkey. *Tohoku Journal of Experimental Medicine*.

[18] Bal AE, Gravekamp C, Hartskeerl RA, De Meza-Brewster J, Korver H, Terpstra WJ (1994). Detection of leptospires in urine by PCR for early diagnosis of leptospirosis. *Journal of Clinical Microbiology*.

